# Research and Development of Zincoborates: Crystal Growth, Structural Chemistry and Physicochemical Properties

**DOI:** 10.3390/molecules24152763

**Published:** 2019-07-30

**Authors:** Yanna Chen, Min Zhang, Miriding Mutailipu, Kenneth R. Poeppelmeier, Shilie Pan

**Affiliations:** 1 CAS Key Laboratory of Functional Materials and Devices for Special Environments, Xinjiang Technical Institute of Physics & Chemistry, CAS; Xinjiang Key Laboratory of Electronic Information Materials and Devices, 40-1 South Beijing Road, Urumqi 830011, China; 2 Center of Materials Science and Optoelectronics Engineering, University of Chinese Academy of Sciences, Beijing 100049, China; 3 Department of Chemistry, Northwestern University, Evanston, IL 60208, USA

**Keywords:** zincoborate, structural chemistry, structure–property relationship, non-linear optical crystal

## Abstract

Borates have been regarded as a rich source of functional materials due to their diverse structures and wide applications. Therein, zincobrates have aroused intensive interest owing to the effective structural and functional regulation effects of the strong-bonded zinc cations. In recent decades, numerous zincoborates with special crystal structures were obtained, such as Cs_3_Zn_6_B_9_O_21_ and AZn_2_BO_3_X_2_ (A = Na, K, Rb, NH_4_; X = Cl, Br) series with KBe_2_BO_3_F_2_-type layered structures were designed via substituting Be with Zn atoms, providing a feasible strategy to design promising non-linear optical materials; KZnB_3_O_6_ and Ba_4_Na_2_Zn_4_(B_3_O_6_)_2_(B_12_O_24_) with novel edge-sharing [BO_4_]^5−^ tetrahedra were obtained under atmospheric pressure conditions, indicating that extreme conditions such as high pressure are not essential to obtain edge-sharing [BO_4_]^5−^-containing borates; Ba_4_K_2_Zn_5_(B_3_O_6_)_3_(B_9_O_19_) and Ba_2_KZn_3_(B_3_O_6_)(B_6_O_13_) comprise two kinds of isolated polyborate anionic groups in one borate structure, which is rarely found in borates. Besides, many zincoborates emerged with particular physicochemical properties; for instance, Bi_2_ZnOB_2_O_6_ and BaZnBO_3_F are promising non-linear optical (NLO) materials; Zn_4_B_6_O_13_ and KZnB_3_O_6_ possess anomalous thermal expansion properties, etc. In this review, the synthesis, crystal structure features and properties of representative zincoborates are summarized, which could provide significant guidance for the exploration and design of new zincoborates with special structures and excellent performance.

## 1. Introduction

Over the past decades, borates have attracted burgeoning attention owning to their excellent properties and wide applications such as non-linear optical (NLO) materials, birefringent materials, electrode materials, etc [1,2,3,4,5,6,7]. The attractive properties of borates mainly depend on their structural diversity, as the boron atoms can be three- or four-coordinated with oxygen atoms to construct the planar triangular [BO_3_]^3−^ groups or tetrahedral [BO_4_]^5−^ groups, respectively, which could further link together via corner- or edge-sharing to form different types of fundamental building blocks (FBBs) [8,9,10,11]. For example, the FBBs of classical KBe_2_BO_3_F_2_ (KBBF) [12,13] and Sr_2_Be_2_B_2_O_7_ (SBBO) [14,15] are [BO_3_]^3−^ triangles, while the commercial NLO crystals *β*-BaB_2_O_4_ (*β*-BBO) [16] and LiB_3_O_5_ (LBO) [17] are composed of [B_3_O_6_]^3−^ and [B_3_O_7_]^5−^ groups, respectively. These FBBs, in turn, may polymerize to form isolated clusters, infinite one-dimensional (1D) chains, two-dimensional (2D) layers or three-dimensional (3D) frameworks, which endow borates with rich structural chemistry and extensive applications [18,19,20,21].

From the viewpoint of structural chemistry, the anionic groups are relatively independent structural and functional modules. The expected excellent optical properties could be achieved through screening and assembling FBBs with a specific arrangement [22,23,24,25,26,27,28], which constitutes a tight combination between the chemistry synthesis and optical properties of materials, as well as establishes a theoretical foundation for rationally designing or synthesizing ultraviolet (UV) and deep-UV (DUV) NLO materials. In particular, the isolated planar B-O groups with coplanar or aligned arrangement could produce large microscopic second-order susceptibility and birefringence, which are suitable to design NLO and birefringent materials for UV/DUV light generation [29,30,31]. For instance, KBBF and *β*-BBO with isolated coplanar B-O units ([BO_3_]^3−^ and [B_3_O_6_]^3−^) exhibit large birefringence and second harmonic generation (SHG) response, which enables them to be world famous as optical crystals and widely applied in the field of laser technology [32,33,34]. However, the design and synthesis of borates with isolated coplanar B-O groups is still a research challenge. According to the statistical analysis of borate FBBs proposed by P. Becker [35], isolated borate polyhedra occur for cations/boron (A:B) > 1, which guides researchers to obtain borates with isolated B-O groups by increasing the ratio of cations and boron [36,37,38]. Recently, many investigations reveal that the introduction of covalent mental cations (such as, Be^2+^, Mg^2+^, Zn^2+^, Al^3+^, etc.) may effectively restrain the polymerization of B-O units and is beneficial to the formation of isolated B-O groups [39,40,41,42,43]. Moreover, the cooperation of highly-coordinated cations (such as, Ba^2+^, Sr^2+^, Rb^+^, K^+^, etc.) with low-coordinated covalent mental cations can synergistically affect the frameworks and facilitate the benign B-O arrangement, such as SBBO, Na_2_Be_4_B_4_O_11_ [44], Cs_3_Zn_6_B_9_O_21_ [45,46], Ba_3_Mg_3_(BO_3_)_3_F_3_ [47], A_3_B_3_Li_2_M_4_B_6_O_20_F (A = K, Rb; B = Ba, Sr; M = Al, Ga) series [48,49,50,51,52,53,54], etc. 

In the last few decades, borates containing zinc cations have become a research focus and zincoborates with novel structures or good properties were continuously reported [55,56,57]. There are several advantages motivating researchers to design new compounds in a zinc-containing borate system: (1) the zinc-containing borate has been regarded as a fertile field to search for the expected compounds with high physicochemical performance. In recent years, using Zn^2+^ to substitute Be^2+^ in the KBBF family has been proved to be a rational design strategy to synthesize desired compounds with KBBF-type layered structures, which is an effective way to explore new UV/DUV NLO materials. For example, KZn_2_BO_3_Cl_2_ [58,59] and Cs_3_Zn_6_B_9_O_21_ were designed with KBBF-type layered structures and exhibit enhanced SHG efficiency of about 3.0 and 3.3 × KH_2_PO_4_ (KDP), respectively. Furthermore, the [ZnO_4_]^6−^ tetrahedron has been regarded as a NLO-active unit with good UV transparency. For instance, Ba_3_(ZnB_5_O_10_)PO_4_ [60], BaZnBO_3_F [61], Ba_5_Zn_4_(BO_3_)_6_ [62], Ba_2_Zn(BO_3_)_2_ [63,64], etc. exhibit enhanced NLO performance. In addition, KZnB_3_O_6_ and Zn_4_B_6_O_13_ possess anomalous thermal expansion properties [65,66,67,68], which revive the studies on new functionalities in borates that have long been overlooked, and might eventually give rise to the discovery of more exciting and exotic emerging physicochemical properties in borates. (2) Many zincoborates possess exceptional structural configurations. For instance, KZnB_3_O_6_ is the first borate possessing the novel edge-sharing (es-) [BO_4_]^5−^ tetrahedra synthesized under ambient pressure [69,70]. Previously, the es-[BO_4_]^5−^ tetrahedra were considered only to exist in some compounds synthesized under high-pressure/high-temperature conditions [71,72,73,74]. Interestingly, Ba_4_Na_2_Zn_4_(B_3_O_6_)_2_(B_12_O_24_) [75] is the second case of borate possessing the es-[BO_4_]^5−^ tetrahedra that obtained under ambient pressure. Besides, several zincoborates comprise of two types of isolated B-O groups, which violates the Pauling’s Rule of parsimony (Pauling’s fifth rule) [76,77]. For instance, Cs_3_Zn_6_B_9_O_21_ ([BO_3_]^3−^ + [B_3_O_6_]^3−^), Ba_3_Zn(BO_3_)(B_2_O_5_)F and Ba_4_Zn_2_(BO_3_)_2_(B_2_O_5_)F_2_ [78] with [BO_3_]^3−^ triangles and another relatively low-polymerized polyborate anions have been reported; moreover, compounds with two kinds of isolated polyborate anions, such as Ba_2_KZn_3_(B_3_O_6_)(B_6_O_13_) [79], Ba_4_K_2_Zn_5_(B_3_O_6_)_3_(B_9_O_19_) [80], Ba_4_Na_2_Zn_4_(B_3_O_6_)_2_(B_12_O_24_), Bi_2_ZnO(B_2_O_6_) ([B_2_O_5_]^4−^ + [B_2_O_7_]^8−^) [81,82,83] and *α*-/*β*-/*γ*-Pb_2_Ba_4_Zn_4_B_14_O_31_ ([B_2_O_5_]^4−^ + [B_6_O_13_]^8−^) [84] were also reported, which are rarely found in borates, implying the special role of zinc cations in regulating and controlling the B-O configuration. 

Herein, the aim of this review is to focus on the crystal chemistry, optical properties, thermal properties and the structure–property relationship of zincoborates due to the significant regulation effects of zinc cations to the structures and properties. We hope that this work could open a way to design novel NLO crystals in zinoborate system, as well as the discovery of new zincoborates with novel structures or particular physicochemical properties.

## 2. Structural Chemistry of Zincoborates

### 2.1. Statistical Analysis of Structural Configurations 

**Figure 1:**
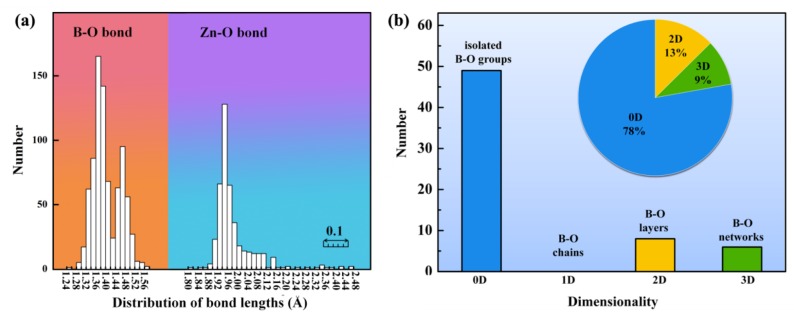
Distribution of (**a**) Zn-O and B-O bond lengths; (**b**) dimensionality of B-O configuration in zinc-containing borates.

In order to better understand the structural diversity of zincoborates, systematic analysis of the Zn-B-O system was carried out by taking the Inorganic Crystal Structure Database (ICSD-4.2.0, the latest release of ICSD-2019/1) as the source of data. The connection modes of zinc cations with oxygen or halogen anions as well as the influence of the zinc cations to the structures are investigated. The thresholds of bond lengths were applied for the Zn-O and B-O bonds at the maximum in the continuity distribution of the Zn-O distances (2.6 Å) and the B-O distances (1.65 Å), respectively. All the available anhydrous and disorder-free zinc-containing borates (64 cases) are summarized. Structural comparisons were carried out and summarized as follows:

(1) The Zn-O, Zn-X (X = F, Cl, Br) bond lengths of the summarized compounds range from 1.814–2.479 Å, 2.023–2.516 Å, respectively, which is in agreement with reasonable values. As shown in Figure 1a, most of the Zn-O bond lengths are in the range of 1.90–2.16 Å. The zinc atoms can coordinate with four, five, or six O/X atoms to form [ZnO_4_]^6−^, [ZnO_5_]^8−^, [ZnO_6_]^10−^, [ZnO_3_X]^5−^, [ZnO_4_X]^7−^, [ZnO_3_X_2_]^6−^ polyhedra, respectively, and the coordination of zinc are mostly four coordinated with oxygen (about 66% of zincoborates contain the [ZnO_4_]^6−^ tetrahedra). These zinc-centered polyhedra can polymerize into isolated Zn-O/X clusters, 1D chains, 2D layers or 3D network through vertex-/edge-sharing, which can further connect with different B-O groups to form Zn-B-O/X structures. To the best of our knowledge, the Zn-B-O configurations in zincoborates are always 2D layers or 3D frameworks, except that the special [Zn_2_(BO_3_)_6_]^14−^ and [ZnB_6_O_18_]^16−^ isolated clusters exist in Ba_2_ZnSc(BO_3_)_3_ [85] and Pb_8_Zn(BO_3_)_6_ [86], respectively. 

(2) The B-O bond lengths of the summarized compounds range from 1.254–1.565 Å, which are in accordance with those of other reported borates. Most of the B-O bond lengths are distributed in the range of 1.30–1.52 Å (Figure 1a). As shown in Figure 1b, approximately 78% of zincoborate structures are built up of isolated B-O groups (0D), such as isolated [BO_3_]^3−^, [B_2_O_5_]^4−^, [B_3_O_6_]^3−^, [B_5_O_10_]^5−^, [B_6_O_12_]^6−^ units, etc., while no 1D B-O configuration is observed. It could be considered that the introduction of zinc cations has profitable impact on the prevention of polymerization of B-O anionic structures. Notably, about 20 % of zincoborates with isolated B-O groups contain coplanar (or nearly coplanar) [BO_3_]^3−^/[B_3_O_6_]^3−^ units, which paves a comprehensive road map for us to design new compounds with benign isolated B-O groups.

### 2.2. Zincoborates Possessing Special Structural Features

It is expected that the introduction of zinc cations into borates can enrich the structural diversity and be beneficial to obtain new zincoborates with special structural features. In this section, several compounds with distinctive crystal structure characteristics are given.

#### 2.2.1. Zincoborates with Benign KBe_2_BO_3_F_2_ (KBBF)-Type Layered Structures

The world-famous KBBF is the unique practical NLO crystal in the DUV region that can generate the 177.3 nm coherent laser by a direct SHG method using Q-switched neodymium (Nd):YAG (1064 nm) laser [87]. Structurally, the perfectly coplanar [BO_3_]^3−^ anionic groups in the ^2^_∞_[Be_2_BO_3_F_2_] layers of KBBF provide a relatively large SHG coefficient (*d*_11_ = 0.47 pm/V) and a moderate birefringence (Δ*n* = 0.07 @1064 nm), which endow KBBF with unprecedented performances [88,89]. The excellence for the KBBF family crystals used as the DUV NLO crystals inspirits researchers to explore beryllium-free borates with benign KBBF-type layered structures [90,91,92,93]. 

To inherit the favorable structural arrangement of KBBF, one effective molecular engineering design strategy is to substitute the [BeO_3_F]^5−^ tetrahedra in ^2^_∞_[Be_2_BO_3_F_2_] layers of KBBF with [MO_4_]/[MO_3_F] (M = Li^+^, Zn^2+^, Al^3+^, and Ga^3+^, etc.) tetrahedra to achieve structural modification [94,95,96]. Therein, most of Zn^−^ and Be-O/X polyhedra have similar coordination environment and approximate bond length, thus the substitution of Zn^2+^ for Be^2+^ has attracted the most attention and obtained a number of compounds possessing benign KBBF-type layered structures, for example, AZn_2_BO_3_X_2_ (A = Na, K, Rb, NH_4_; X = Cl, Br) series [58,59], Cs_3_Zn_6_B_9_O_21_ [45,46], BaLiZn_3_(BO_3_)_3_ [97,98], CdZn_2_KB_2_O_6_F [99,100], etc. 

##### AZn_2_BO_3_X_2_ (A = Na, K, Rb, NH_4_; X = Cl, Br) Series with ^2^_∞_[Zn_2_BO_3_X_2_] Layers

**Figure 2:**
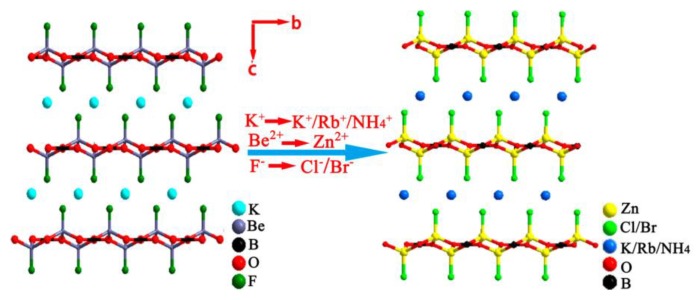
Structural evolution from KBBF to AZn_2_BO_3_X_2_. Reprinted with permission from Ref. [58]. Copyright (2016) American Chemical Society.

AZn_2_BO_3_X_2_ (A = Na, K, Rb, NH_4_; X = Cl, Br) series of compounds were reported by two independent groups of Chen and Ye in 2016 [58,59], which were developed as the production of the “transgenosis” process on KBBF structure, specifically, using [ZnO_3_X]^5−^ tetrahedra to substitute the [BeO_3_F]^5−^ tetrahedra and yield the ^2^_∞_[Zn_2_BO_3_X_2_] layers similar to the ^2^_∞_[Be_2_BO_3_F_2_] layers in KBBF (Figure 2). AZn_2_BO_3_X_2_ (A = Na, K, Rb, NH_4_; X = Cl, Br) crystals all crystallize in *R*32 (No. 155) chiral space group and are isostructural to KBBF. In the ^2^_∞_[Zn_2_BO_3_X_2_] layers, the [ZnO_3_X]^5−^ tetrahedra induce [BO_3_]^3−^ groups to arrange into a nearly coplanar and aligned arrangement, which is beneficial to generate large SHG responses and birefringence, indicating that they are likely to inherit the optical advantages of KBBF. 

##### Cs_3_Zn_6_B_9_O_21_ with ^2^_∞_[Zn_2_BO_3_O_2_] Layers

**Figure 3:**
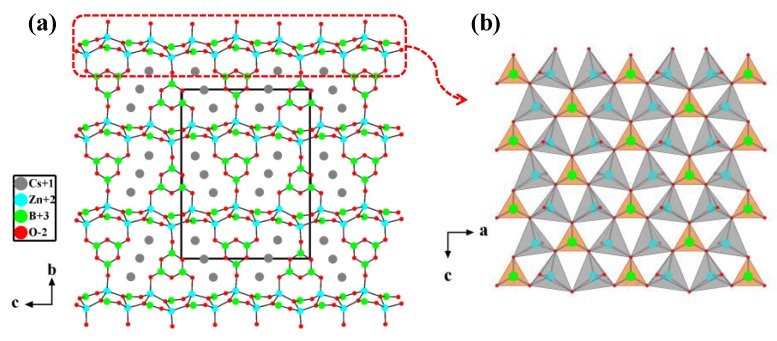
(**a**) The crystal structure of Cs_3_Zn_6_B_9_O_21_ viewed along the *a*-axis; (**b**) the ^2^_∞_[Zn_2_BO_3_O_2_] layer composed of [ZnO_4_]^6−^ tetrahedra and nearly coplanar [BO_3_]^3−^ triangles. Adapted with permission from Ref. [45,46]. Copyright (2014) American Chemical Society.

On the basis of substituting Be^2+^ with Zn^2+^, Cs_3_Zn_6_B_9_O_21_ with ^2^_∞_[Zn_2_BO_3_O_2_] layers was synthesized and reported by two independent groups as a new UV NLO material [45,46]. Cs_3_Zn_6_B_9_O_21_ crystallizes in the orthorhombic system of space group *Cmc*2_1_ (No. 36). Within the targeted ^2^_∞_[Zn_2_BO_3_O_2_] layers, the [BO_3_]^3−^ triangles are in an approximately coplanar and aligned arrangement, which is arranged by the [ZnO_4_]^6−^ tetrahedra (Figure 3b). The adjacent ^2^_∞_[Zn_2_BO_3_O_2_] layers are bridged through [B_3_O_6_]^3−^ groups (Figure 3a), which would reinforce the interlayer bonding compared with the weak K^+^-F^−^ ionic bonds in KBBF. Verified by experiments, Cs_3_Zn_6_B_9_O_21_ maintains the optical properties of KBBF, and the crystals of Cs_3_Zn_6_B_9_O_21_ are of block shape without layering tendency. 

##### BaLiZn_3_(BO_3_)_3_ with ^2^_∞_[LiZn_3_(BO_3_)_3_] Layers and CdZn_2_KB_2_O_6_F with ^2^_∞_[ZnBO_3_] Layers

**Figure 4:**
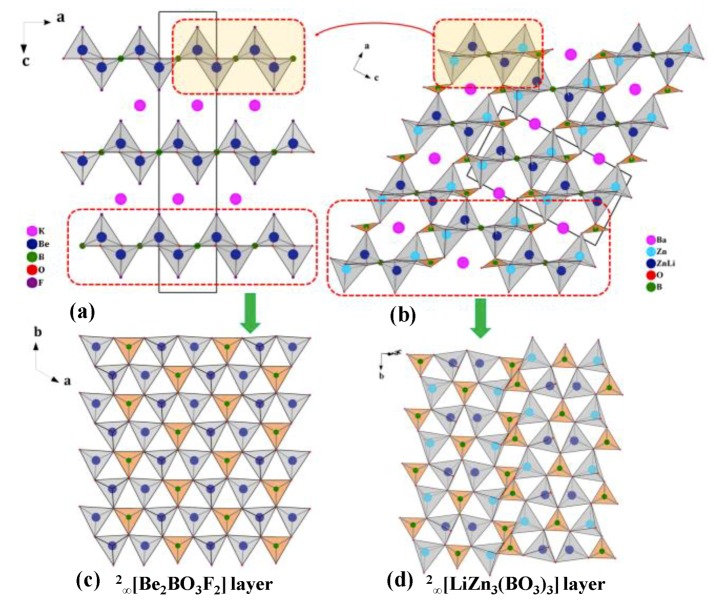
The crystal structures of (**a**) KBe_2_BO_3_F_2_ (KBBF) and (**b**) BaLiZn_3_(BO_3_)_3_ viewed along the *b*-axis; (**c**) The ^2^_∞_[Be_2_BO_3_F_2_] layer in KBBF and (**d**) the ^2^_∞_[LiZn_3_(BO_3_)_3_] layer in BaLiZn_3_(BO_3_)_3_. [97]-Reproduced by permission of The Royal Society of Chemistry (RSC) on behalf of the Centre National de la Recherche Scientifique (CNRS) and the RSC.

BaLiZn_3_(BO_3_)_3_ [97,98] and CdZn_2_KB_2_O_6_F [99,100] are the other two beryllium-free borates with KBBF-type structures. BaLiZn_3_(BO_3_)_3_ features a special *zigzag* ^2^_∞_[LiZn_3_(BO_3_)_3_] layer that is constructed by ^1^_∞_[LiZn_3_O_11/3_(BO_3_)] chains and [BO_3_]^3−^ units, which is evolved from ^2^_∞_[Be_2_BO_3_F_2_] layer in KBBF (Figure 4). The adjacent ^2^_∞_[LiZn_3_(BO_3_)_3_] layers are tightly stacked directly via Li/Zn-O bonds in the layers, which is different from most of the KBBF derivatives that connect the adjacent layers through cations or B-O groups between the layers. For instance, the adjacent layers in KBBF [12,13], Na_2_CsBe_6_B_5_O_15_ [101], *β*/*γ*-KBe_2_B_3_O_7_ [102] and Cs_3_Zn_6_B_9_O_21_ [45,46], are connected by K-F bonds, [BO_3_]^3−^ groups, ^1^_∞_[BO_2_] chains and [B_3_O_6_]^3−^ groups, respectively. The strong Li/Zn-O covalent bonds in BaLiZn_3_(BO_3_)_3_ can effectively reinforce the interlayer force and improve the layering tendency of KBBF-type structures. 

**Figure 5:**
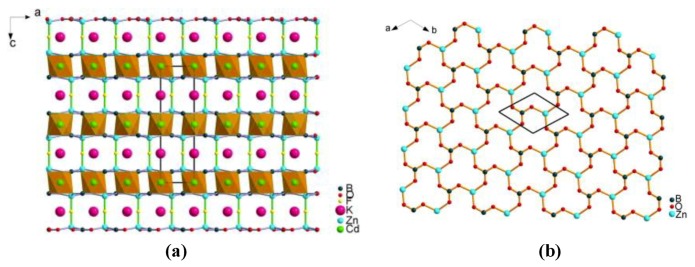
(**a**) The crystal structure of CdZn_2_KB_2_O_6_F viewed along the *b*-axis; (**b**) the single ^2^_∞_[ZnBO_3_] layer. Reprinted from Ref. [99], Copyright (2009) with permission from Elsevier.

CdZn_2_KB_2_O_6_F crystallizes in the space group of *P*$\bar{3}$1*c* (No. 163) [99,100]. In the structure, the [BO_3_]^3−^ triangles and [ZnO_3_]^4−^ pyramids (from the [ZnO_3_F]^5−^ tetrahedra) share the vertex O atoms to form a ^2^_∞_[ZnBO_3_] layer (Figure 5b), which is also similar to the ^2^_∞_[Be_2_BO_3_F_2_] layer in KBBF. The ^2^_∞_[ZnBO_3_] layers are connected by bridging F and Cd atoms alternately along the *c*-axis, and the K^+^ cations are filled in the interlayer to balance charge (Figure 5a). In the ^2^_∞_[ZnBO_3_] layers, the [BO_3_]^3−^ triangles are also in a coplanar arrangement influenced by the [ZnO_3_F]^5−^ tetrahedra (Figure 5b). 

#### 2.2.2. Zincoborates with Novel Edge-Sharing [BO_4_]^5−^ Tetrahedra

On the basis of the borate structures discovered, the [BO_4_]^5−^ units usually connect to each other via corner-sharing (cs-) rather than edge-sharing (es-) or face-sharing [103,104,105]. In terms of Pauling’s 3rd and 4th rules and the orbital interpretation rules, the connection mode of es-polyhedra for high-valence and low coordinated small cations is scarcely seen except under extreme conditions such as high pressure (HP), for the reason that the repulsion interactions between the adjacent cations and anions may be increased when two anion-based polyhedra adopt edge-sharing connection mode [76,77]. Thus, the formation of es-[BO_4_]^5−^ tetrahedra is extremely unfavored, and, the es-[BO_4_]^5−^ units can only be observed in very few borates. 

In 2002, borate with es-[BO_4_]^5−^ tetrahedra, Dy_4_B_6_O_15_ [106], was firstly synthesized under high pressure conditions (8 GPa, 1000 K) by Huppertz and van der Eltz, which indicates that the [BO_4_]^5−^ tetrahedra can link together not only via common corners but also via common edges. Since then, several new es-[BO_4_]^5−^ tetrahedra-containing borates have been synthesized under high pressure and high temperature conditions, for instance, RE_4_B_6_O_15_ (RE = Dy and Ho) [107], *α*-(RE)_2_B_4_O_9_ (RE = Eu, Gd, Tb, Dy, Sm, Ho) [108,109], HP-AB_3_O_5_ (A = K, NH_4_, Rb, Tl) [71,72,73] and HP-MB_2_O_4_ (M = Fe, Ni, Co) [110,111]. Recently, *α*-Ba_3_[B_10_O_17_(OH)_2_] with es-[BO_4_]^5−^ tetrahedra was synthesized through hydrothermal reactions at 500 °C and 1000 bar [112]. Although the es-[BO_4_]^5−^ tetrahedra appear in these compounds, the high pressure condition is indispensable prerequisite. Extraordinarily, two zincoborates, KZnB_3_O_6_ [69,70] and Ba_4_Na_2_Zn_4_(B_3_O_6_)_2_(B_12_O_24_) [75], possessing es-[BO_4_]^5−^ configuration were obtained under ambient pressure in 2010 and 2013, respectively, demonstrating that high pressure is not essential for the formation of es-[BO_4_]^5−^ polyhedra. In addition, the synthesis of Li_4_Na_2_CsB_7_O_14_ and BaAlBO_4_ with es-[BO_4_]^5−^ tetrahedra further enriches the es-[BO_4_]^5−^ containing borate system synthesized under atmospheric environment [113,114]. Very recently, *β*-CsB_9_O_14_, the first triple-layered borate with es-[BO_4_]^5−^ tetrahedra, was obtained under the vacuum sealed condition [115].

##### KZnB_3_O_6_

**Figure 6:**
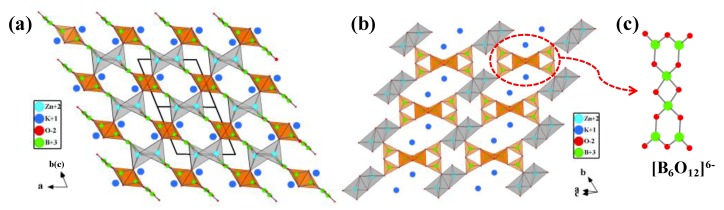
(**a**) The crystal structure of KZnB_3_O_6_ viewed along the [01$\bar{1}$] direction; (**b**) Connection details of [B_6_O_12_]^6−^ and [Zn_2_O_6_]^8−^ blocks in the ($\bar{1}$ 11) plane; (**c**) The [B_6_O_12_]^6−^ group. Adapted from Ref. [66,69], with permission of John Wiley and Sons.

KZnB_3_O_6_ crystallizes in the space group of *P*$\bar{1}$ (No. 2) [69,70]. As shown in Figure 6, its structure contains a remarkable [B_6_O_12_]^6−^ group consisting of two es-[BO_4_]^5−^ tetrahedra and four cs-[BO_3_]^3−^ triangles, the [B_6_O_12_]^6−^ groups are further connected by distorted [ZnO_4_]^6−^ tetrahedra in edge-shared pairs to form a 3D framework, then the K^+^ cations fills in the cavities to construct the whole structure.

KZnB_3_O_6_ was synthesized using a conventional solid-state reaction under ambient pressure. In detail, a single-phase white powder of KZnB_3_O_6_ was prepared by grinding a stoichiometric mixture of K_2_CO_3_, ZnO, and H_3_BO_3_, which was heated to 500 °C to decompose the salt and annealed at 750 °C for 24 h. Single crystals of KZnB_3_O_6_ were obtained by spontaneous nucleation by melting the obtained pure phase powder at 820 °C, then slowly cooling the melt to 600 °C at a rate of 1 °C h^−1^. Although the synthesis condition of KZnB_3_O_6_ is different from that of previously reported HP borates, further examination of the edge-sharing geometry reveals that the B-O bond lengths and O-B-O angles in KZnB_3_O_6_ are consistent with those of HP borates. As a common feature, the O-B-O angles within the [B_2_O_2_] ring of es-[BO_4_]^5^^−^ are remarkably reduced and the B-O bonds within the ring are elongated due to the like-charges repulsion, which will push higher valence ions apart in the es-polyhedra to minimize the electrostatic potential.

Theoretical insight into the structural stability of KZnB_3_O_6_ was carried out by Yang and coworkers [116]. They investigated the molecular dynamics, lattice dynamics and electronic properties of es-KZnB_3_O_6_ and cs-KZnB_3_O_6_ (hypothetical one, constructed based on isostructural KCdB_3_O_6_) via density functional theory. Molecular dynamics simulations show that, es-KZnB_3_O_6_ is stable from 100 to 1000 K while cs-KZnB_3_O_6_ deforms with bond stretching. Analysis of lattice dynamics shows that, a soft-mode reflecting the dynamic instability exists in the cs-KZnB_3_O_6_, which probably comes from an overlong Zn-O bond in the [ZnO_5_]^8−^ polyhedra. Electronic property calculation indicates that the longest B-O *σ* bonds connecting the es-[BO_4_]^5−^ polyhedra are stable enough to provide a solid framework for es-KZnB_3_O_6_. The stability of cs-KZnB_3_O_6_ is reduced by the overlong Zn-O bond that possesses the smallest covalent nature and the least orbital overlap among the bonds in a [ZnO_5_]^8−^ polyhedron, which further confirms the results that are obtained from lattice dynamics analysis. The results strongly support explanation of the structural stability origination of es-KZnB_3_O_6_, and provide a fundamental understanding on the origin of the unique es-[BO_4_]^5−^ connection mode.

##### Ba_4_Na_2_Zn_4_(B_3_O_6_)_2_(B_12_O_24_)

**Figure 7:**
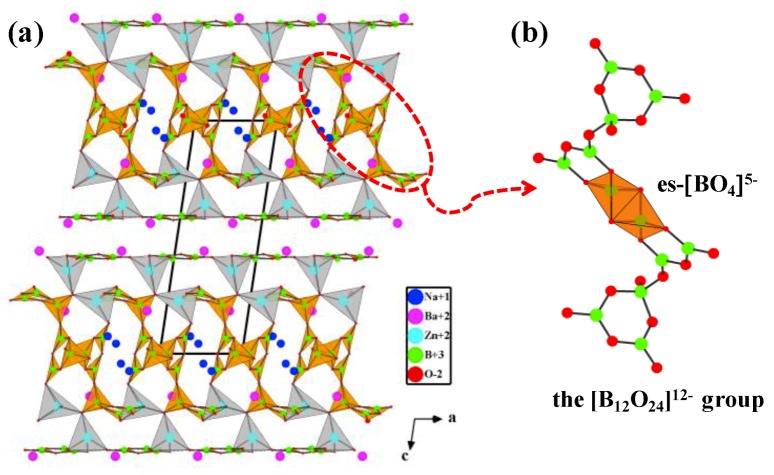
(**a**) The crystal structure of Ba_4_Na_2_Zn_4_(B_3_O_6_)_2_(B_12_O_24_) viewed along the *b*-axis; (**b**) the [B_12_O_24_]^12−^ group containing es−[BO_4_]^5−^ tetrahedra. Adapted from Ref. [75], Copyright (2013) with permission from Elsevier.

Ba_4_Na_2_Zn_4_(B_3_O_6_)_2_(B_12_O_24_) is the second reported borate possessing the special es-[BO_4_]^5−^ configuration obtained under ambient pressure [75]. Single crystals of Ba_4_Na_2_Zn_4_(B_3_O_6_)_2_(B_12_O_24_) were synthesized by high temperature solution method using Na_2_CO_3_, BaCO_3_, ZnO, H_3_BO_3_, Na_2_B_4_O_7_⋅10H_2_O as raw materials (Na_2_CO_3_/BaCO_3_/ZnO/H_3_BO_3_/Na_2_B_4_O_7_ molar ratio = 1:4:6:18:2). The basic structural units in Ba_4_Na_2_Zn_4_(B_3_O_6_)_2_(B_12_O_24_) are the [ZnO_4_]^6−^ tetrahedra, [B_3_O_6_]^3−^ and [B_12_O_24_]^12−^ groups. Therein, the [B_12_O_24_]^12−^ group is composed of [BO_3_]^3−^ triangles and [BO_4_]^5−^ tetrahedra via vertex- and edge-sharing. In detail, one [BO_4_]^5−^ tetrahedron and two [BO_3_]^3−^ triangles form a [B_3_O_7_]^5−^ group via vertex-sharing, a [BO_4_]^5−^ tetrahedron of the [B_3_O_7_]^5−^ group links to a [BO_3_]^3−^ triangle of another [B_3_O_7_]^5−^ group to form a [B_6_O_13_]^8−^ group, then two inversion-center-related [B_6_O_13_]^8−^ groups are further connected by es-[BO_4_]^5−^ tetrahedra to form a [B_12_O_24_]^12−^ group (Figure 7b). As shown in Figure 7a, the [B_12_O_24_]^12−^ groups are located between parallel [B_3_O_6_]^3−^ rings to form a sandwich structural block, which are bridged by [ZnO_4_]^6−^ tetrahedra to generate a 2D infinite ^2^_∞_[Zn_4_(B_3_O_6_)_2_(B_12_O_24_)] layer. The intralayer open channels and interlayer void spaces are filled with Ba^2+^ and Na^+^ cations to balance charge and form a 3D framework. 

**Figure 8:**
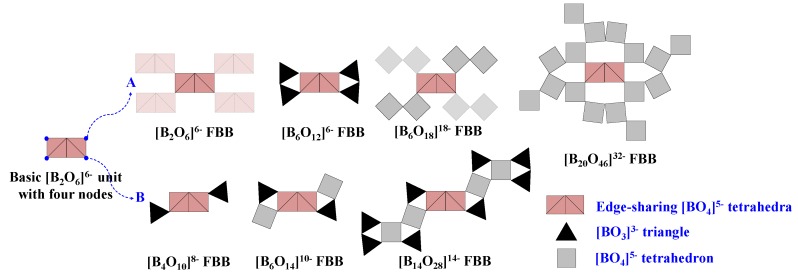
Structural features of all the available anhydrous borates with fundamental building blocks (FBB) containing edge-sharing [BO_4_]^5^^−^ tetrahedra. Adapted from Ref. [113] with permission from The Royal Society of Chemistry.

The structural features of es-[BO_4_]^5−^ tetrahedra in aforementioned anhydrous borates are highly consistent and the FBBs share common features. Taking the basic [B_2_O_6_]^6^^−^ unit as the prototype, all the available FBBs of these es-[BO_4_]^5−^ tetrahedra-containing borates can be evolved by replacing the four nodes with different types of B-O blocks (Figure 8). For the type A model, the replaced nodes are the same B-O blocks, such as the [B_2_O_6_]^6^^−^ FBB of HP-MB_2_O_4_, [B_6_O_12_]^6^^−^ FBB of KZnB_3_O_6_, [B_6_O_18_]^18^^−^ FBB of RE_4_B_6_O_15_, and [B_20_O_46_]^32^^−^ FBB of a-RE_2_B_4_O_9_ series can be regarded as the derivatives obtained by replacing the nodes of basic [B_2_O_6_]^6^^−^ units with four identical [B_2_O_6_]^6^^−^, [BO_3_]^3^^−^, [B_2_O_7_]^8^^−^, and [B_4_O_13_]^14^^−^ units, respectively. While the replaced nodes for type B model are different, such as the corresponding replaced units are [BO_3_]^3^^−^ for [B_4_O_10_]^8^^−^ FBB of BaAlBO_4_, [BO_3_]^3^^−^ and [BO_4_]^5^^−^ for [B_6_O_14_]^10^^−^ FBB of HP-AB_3_O_5_ (A = K, NH_4_, Rb, Tl), as well as [BO_3_]^3^^−^ and [B_5_O_11_]^7^^−^ blocks for [B_14_O_28_]^14^^−^ FBB of Li_4_Na_2_CsB_7_O_14_. 

The above findings prove that the borate structure is very flexible and confirm the feasibility of incorporating the es-[BO_4_]^5−^ configuration into traditional borate chemistry to enrich the borate structure.

#### 2.2.3. Zincoborates with Two Kinds of Isolated Anion Groups

According to the Pauling’s fifth rule [76,77], the number of essentially different kinds of constituents in a crystal tends to be small, which means that the number of components of various types in a crystal tends to be small. For most borates, there is only one kind of isolated B-O group in the structure [117,118]. Although violating Pauling’s fifth rule, several zincoborates with two kinds of isolated B-O groups have been discovered, for instance, Cs_3_Zn_6_B_9_O_21_ ([BO_3_]^3−^ + [B_3_O_6_]^3−^) [45,46], Ba_3_Zn(BO_3_)(B_2_O_5_)F and Ba_4_Zn_2_(BO_3_)_2_(B_2_O_5_)F_2_ [78], etc., in which two kinds of isolated B-O groups are [BO_3_]^3−^ triangle and another relatively low-polymerized polyborate anion. Specifically, there are few examples of two kinds of isolated polyborate (polymerization is no less than 2) anions coexisting in one zincoborate structure, such as, Ba_2_KZn_3_(B_3_O_6_)(B_6_O_13_) [79], Ba_4_K_2_Zn_5_(B_3_O_6_)_3_(B_9_O_19_) [80], Ba_4_Na_2_Zn_4_(B_3_O_6_)_2_(B_12_O_24_) [75], Bi_2_ZnO(B_2_O_6_) ([B_2_O_5_]^4−^ + [B_2_O_7_]^8−^) [81,82,83], *α*-/*β*-/*γ*-Pb_2_Ba_4_Zn_4_B_14_O_31_ ([B_2_O_5_]^4−^ + [B_6_O_13_]^8−^) [84], etc. The uncommon coexistence of different B-O polyanions in these zincoborates further implies the prevention effect of strong-bonded zinc cations on the polymerization of B-O configuration. 

## 3. Zincoborates with Excellent Properties

Based on the previous reports, it should be emphasized that Zn-O/F polyhedra, especially the [ZnO_4_]^6−^ and [ZnO_3_F]^5−^ tetrahedra, have impacts on both crystal structures and properties. In this section, a series of zincoborates with UV/DUV cutoff edges, second-order NLO properties and anomalous thermal expansion properties are briefly reviewed.

### 3.1. Zincoborates with Short Ultraviolet (UV) Cutoff Edges

With the rapid development of UV technology, NLO and birefringent materials with high transparency in the UV regions are generally required from both an academic and technological standpoint [119,120,121]. Since the *d*-*d* or *f*-*f* electronic transitions will have a negative influence on the large energy band gap, thus, in consideration of the absorption edge, it is a common strategy to introduce cations without *d*-*d* or *f*-*f* transitions (such as alkali and alkaline-earth metals) to blue shift the cutoff edge to the UV regions [122,123]. Besides, cations with fully occupied *d* or half-occupied *f* electronic shells, such as Zn^2+^, Gd^3+^, and Y^3+^, can also be used in UV materials since their electronic shells can effectively inhibit unfavorable electronic transitions [124,125,126,127]. Insofar as we know, there are a number of zinc-containing borates reported in the UV/DUV regions. For instance, Ba_3_(ZnB_5_O_10_)PO_4_ (~180 nm) [60], Cs_3_Zn_6_B_9_O_21_ (~200 nm) [45,46], AZn_2_BO_3_X_2_ (A = Na, K, Rb, NH_4_; X = Cl, Br) series (~190-209nm) [58,59], K_7_ZnSc_2_B_15_O_30_ (~200 nm) [128], K_3_ZnB_5_O_10_ (~190 nm) [129], Cs_12_Zn_4_(B_5_O_10_)_4_ (below 185 nm) [130], etc. Hence, the Zn-containing borate system is also a candidate for exploring promising UV even DUV materials. 

### 3.2. Zincoborates with Large Second-Order Non-Linear Optical (NLO) Response

The increasing need for high-power all-solid-state UV light sources promotes the development of NLO borate crystals, especially those with large second-order NLO responses and short UV-transmission cutoff edges [131,132]. After continuous efforts in the past few decades, a series of borate-based NLO materials were developed and have been widely used in many optoelectronic devices, such as KBBF [12,13], *β*-BBO [16], LBO [17], CsLiB_6_O_10_ (CLBO) [133,134], etc. Up to now, many NLO zincoborates with good performance in the UV/DUV regions have been discovered. In this section, we focus on recent studies of zincoborate crystals with good second-order NLO properties and the representative ones are included in Table 1.

#### 3.2.1. NLO Properties of Zincoborates Containing Alkali/Alkaline-Earth Metals

##### Cs_3_Zn_6_B_9_O_21_

**Figure 9:**
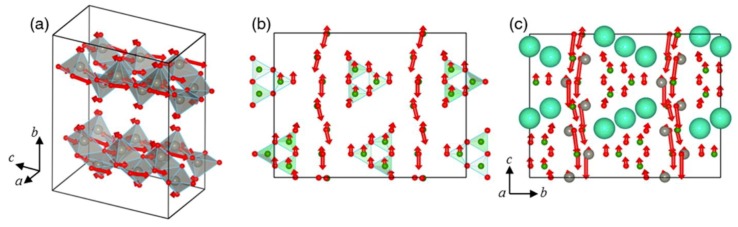
Atomic distortion patterns obtained from the symmetry-mode analysis: Oxygen atom displacements belonging to (**a**) the [ZnO_4_]^6−^ tetrahedra, (**b**) the [BO_3_]^3−^ network, and (**c**) the complete distortion projected along the *a*-axis. Reprinted (adapted) with permission from Ref. [45], Copyright (2014) American Chemical Society.

Single crystals of Cs_3_Zn_6_B_9_O_21_ were grown by the high temperature solution method using Cs_2_O-B_2_O_3_-PbO (Cs_2_CO_3_:ZnO:H_3_BO_3_:PbO = 1:1:5:1) or Cs_2_O-ZnF_2_-B_2_O_3_ (Cs_2_CO_3_:ZnO:ZnF_2_:H_3_BO_3_ = 1.5:1:2:8) as the flux system [45,46]. The absorption edge of Cs_3_Zn_6_B_9_O_21_ is below 200 nm in the UV region and its powder SHG efficiency is approximately 3.3 times that of KDP, which implies that Cs_3_Zn_6_B_9_O_21_ has potential application prospects as an UV NLO material. Remarkably, Cs_3_Zn_6_B_9_O_21_ has a small density of the [BO_3_]^3−^ triangles but exhibits a large SHG response in the KBBF family. Based on the calculation of the dipole moments, the inversion symmetry lifting atomic distortions (Figure 9), electronic structure and atom-cutting analysis, the enhanced SHG response originates from the cooperative effect of coparallel [BO_3_]^3−^ triangles and distorted [ZnO_4_]^6−^ tetrahedra in the ^2^_∞_[Zn_2_BO_3_O_2_] layers. In particular, the contribution of the [ZnO_4_]^6−^ groups to the SHG effect is significantly larger than that from the aligned [BO_3_]^3−^ groups, i.e., the [ZnO_4_]^6−^ tedrahedra dominate the SHG enhancement, which distinguishes [ZnO_4_]^6−^ tedrahedra as an UV-transparent NLO-active units. The results imply that facile synthesis of useful NLO crystals can be achieved by combining [ZnO_4_]^6−^ tetrahedra and π-orbital systems in borates. 

##### AZn_2_BO_3_X_2_ (A = Na, K, Rb, NH_4_; X = Cl, Br) Series

Crystals of KZn_2_BO_3_Cl_2_, RbZn_2_BO_3_Cl_2_, KZn_2_BO_3_Br_2_, and RbZn_2_BO_3_Br_2_ can be obtained by high temperature solution method as well as solvothermal techniques, while crystals of NH_4_Zn_2_BO_3_Cl_2_ were grown only by solvothermal techniques due to the decomposition of ammonium compounds at high temperature. The series of borates are isostructural with KBBF and preserve the NLO-favorable structural features [58,59]. Remarkably, this series of materials exhibits strong SHG responses of approximately more than 2 times that of benchmark KBBF, and the compounds are phase-matchable in the visible and UV regions and possess UV-transmission cutoff edges (~200 nm), indicating that this series of crystals may have potential application in the short-wave NLO field. Theoretical calculations reveal that the SHG enhancement mainly originates from the distorted [ZnO_3_X]^5−^ tetrahedra. The cooperative effect of [ZnO_3_X]^5−^ tetrahedra and the coparallel [BO_3_]^3−^ triangles results in the large SHG responses. In particular, it is the first case where [ZnO_3_X]^5−^ groups can be used as the NLO-active structural units in NLO materials.

##### BaZnBO_3_F

**Figure 10:**
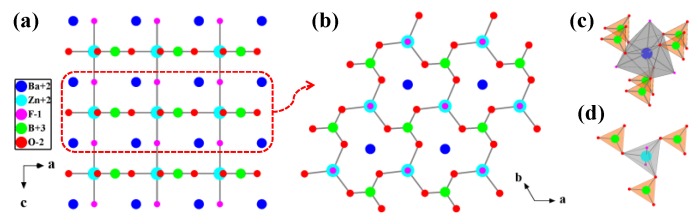
(**a**) Crystal structure of BaZnBO_3_F viewed along the *b*-axis; (**b**) Flat ^2^_∞_[ZnBO_3_F] layer; (**c**) [BaO_6_F_3_]^13−^ polyhedron connects to six [BO_3_]^3−^ groups; (**d**) [ZnO_3_F_2_]^6−^ trigonal bipyramid connects to three [BO_3_]^3−^ groups. Adapted from Ref. [29], Copyright (2018) with permission from Elsevier.

Initially, structure of BaZnBO_3_F was determined by powder X-ray diffraction data in 2010. Single crystal growing trials of BaZnBO_3_F with different fluxes have not been successful until 2016 [61,135]. In the structure of BaZnBO_3_F, the [ZnO_3_F_2_]^6−^ bipyramid shares its three equatorial oxygen atoms with three [BO_3_]^3−^ groups to form a flat ^2^_∞_[ZnBO_3_F] layer, and the adjacent layers are further linked via the apical F atoms of [ZnO_3_F_2_]^6−^ bipyramids to form a 3D framework (Figure 10). Within a single ^2^_∞_[ZnBO_3_F] layer, the [ZnO_3_F_2_]^6−^ bipyramid facilitates its three neighboring [BO_3_]^3−^ units to arrange into a perfect coplanar alignment in the plane through three basal or equatorial bonds of [ZnO_3_F_2_]^6−^ bipyramid. Meanwhile, among different layers, the [BO_3_]^3−^ units are also governed by the [BaO_6_F_3_]^13−^ polyhedra and arranged parallel to each other in neighboring layers. The perfectly coplanar manner of the [BO_3_]^3−^ groups produces a cooperative effect and gives maximum contribution to the NLO response. As a result, a large NLO effective coefficient, 2.8 × *d*_eff_ (KDP), is observed.

##### Ba_5_Zn_4_(BO_3_)_6_

**Figure 11:**
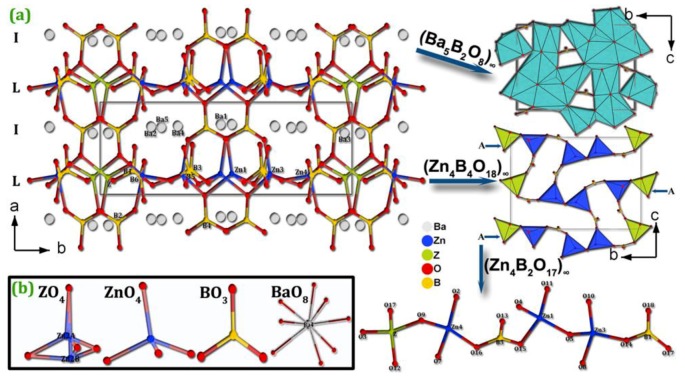
(**a**) The crystal structure of Ba_5_Zn_4_(BO_3_)_6_; (**b**) Basic building blocks in Ba_5_Zn_4_(BO_3_)_6_. Reprinted with permission from Ref. [62], Copyright (2017) American Chemical Society.

A new NLO crystal Ba_5_Zn_4_(BO_3_)_6_ was obtained by substituting the Be atom with the Zn atom, single crystals of which were obtained from a high-temperature solution with BaCO_3_, ZnO, H_3_BO_3_, and NaF in a molar ratio of 2:2:4:1 [62]. The structure is constructed with ^2^_∞_[Zn_4_(BO_3_)_4_O_6_] layers bridged by planar [BO_3_]^3−^ groups (Figure 11), and the distance between adjacent ^2^_∞_[Zn_4_(BO_3_)_4_O_6_] layers in Ba_5_Zn_4_(BO_3_)_6_ is much shorter than that of KBBF, thus, Ba_5_Zn_4_(BO_3_)_6_ may show a better growth habit. Also, Ba_5_Zn_4_(BO_3_)_6_ features a relatively large SHG response of about 2.6 times that of KDP, owing to the incorporation of [BO_3_]^3−^ and [ZnO_4_]^6−^ NLO active groups. Calculation results, based on the anion group theory [138,139], show that the theoretically calculated SHG response coming from the [BO_3_]^3−^ groups (*d*_111_ and *d*_122_ coefficients with values of +0.403 and −0.42 pm/V, respectively) is far smaller than the total SHG response, which implies that the [ZnO_4_]^6−^ groups contribute a lot to the large SHG response, and further confirms the function of the [ZnO_4_]^6−^ groups as NLO active groups. 

#### 3.2.2. Other Zinc-Containing Compounds with NLO Properties

##### Bi_2_ZnOB_2_O_6_

**Figure 12:**
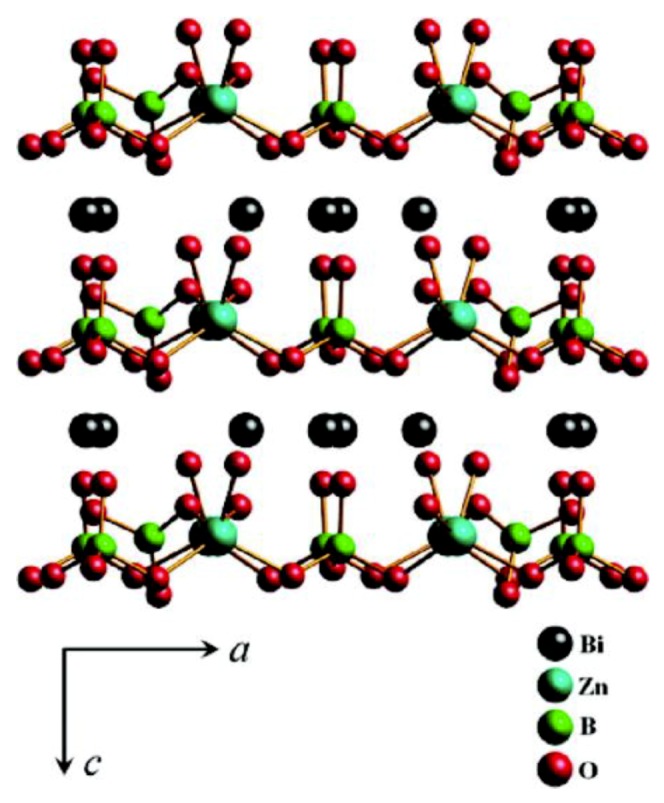
The crystal structure of Bi_2_ZnOB_2_O_6_ viewed along *b*-axis. Reprinted with permission from Ref. [140], Copyright (2009) American Chemical Society.

Bi_2_ZnOB_2_O_6_ was first reported by J. Barbier et al. in 2005 and its structure was determined by powder X-ray diffraction and refined by the Rietveld method using powder neutron diffraction data [81]. Two years later, the crystal of Bi_2_ZnOB_2_O_6_ with a size of 0.4 × 0.4 × 0.5 mm^3^ was prepared by the conventional solid-state reaction method [82]. Until 2009, Pan group firstly obtained the high quality sizable single crystal by the top-seeded growth method [83]. The structure of Bi_2_ZnOB_2_O_6_ consists of ^2^_∞_[ZnB_2_O_7_] layers alternating with six-coordinated Bi^3+^ cations (Figure 12) [140]. In the ^2^_∞_[ZnB_2_O_7_] layer, [B_2_O_5_]^4^^−^ and [B_2_O_7_]^8^^−^ units are bridged by [ZnO_4_]^6^^−^ tetrahedra via sharing oxygen atoms. It is a positive biaxial optical crystal with large birefringence (0.1066-0.0794) and has a large SHG effect of about 3–4 times that of KDP. These advantages make Bi_2_ZnOB_2_O_6_ a promising candidate for NLO materials and attractive for continued research.

##### Ba_3_(ZnB_5_O_10_)PO_4_

**Figure 13:**
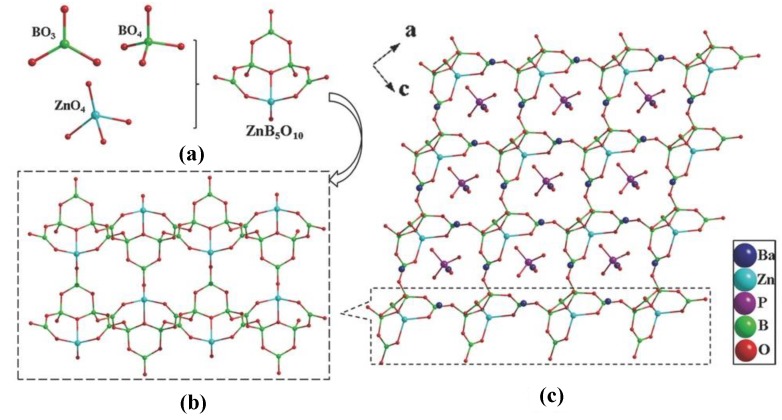
The crystal structure of Ba_3_(ZnB_5_O_10_)PO_4_. (**a**) The [ZnB_5_O_10_]^3−^ units consist of [BO_3_]^3−^, [BO_4_]^5−^, and [ZnO_4_]^6−^ polyhedra; (**b**) The ^3^_∞_[ZnB_5_O_10_] network within which the (**c**) [PO_4_]^3−^ anions and Ba^2+^ cations occupy the channels. Reprinted from Ref. [60], with permission of John Wiley and Sons.

Ba_3_(ZnB_5_O_10_)PO_4_ was successfully synthesized as the first DUV NLO zincoborate-phosphate crystal by combining [ZnO_4_]^6−^ tetrahedra, [PO_4_]^3−^ tetrahedra, and B-O groups into one compound [60]. In the crystal structure, the basic building unit [ZnB_5_O_10_]^3−^ is composed of three [BO_3_]^3−^ triangles, two [BO_4_]^5−^ tetrahedra, and one [ZnO_4_]^6−^ tetrahedron via sharing the corner oxygen atoms (Figure 13a). The adjacent [ZnB_5_O_10_]^3−^ building units are further interconnected through corners to create a ^3^_∞_[ZnB_5_O_10_] framework (Figure 13b). The Ba atoms and the [PO_4_]^3−^ tetrahedra are embedded in the voids of the ^3^_∞_[ZnB_5_O_10_] framework (Figure 13c). Ba_3_(ZnB_5_O_10_)PO_4_ exhibits a DUV absorption edge of 180 nm, large SHG responses of approximately 4 × KDP at 1064 nm, and is type-I phase-matchable. All these results indicate that Ba_3_(ZnB_5_O_10_)PO_4_ is a promising NLO material. Based on the calculation results, the rotation of the B-O groups is pivotal for enabling the SHG. In particular, the valence band maximum consists of Zn 3*d* states of O 2*p* states derived from the [ZnO_4_]^6−^ tetrahedra, which along with the [BO_3_]^3−^ triangles make the most important contributions to the NLO response. 

### 3.3. Zincoborates with Anomalous Thermal Expansion Properties

Most of the materials exhibit positive thermal expansion, i.e., expanding on heating and contracting on cooling in three dimensions. Interestingly, an increasing quantity of materials with anomalous thermal expansion properties, such as negative thermal expansion (NTE) (materials contract along some specific directions when heated) and zero thermal expansion (ZTE) (materials can retain a constant size in a specified temperature range), have attracted a great deal of attention in laboratories and industries [141,142].

As abundant inorganic compounds resources, borates not only have promising applications as optical materials, but also are recognized with unusual thermal expansion behavior [143,144]. The bond lengths and angles of [BO_3_]^3−^ triangles or [BO_4_]^5−^ tetrahedra in a borate structure remain almost constant as the ambient temperature varies. When these rigid B-O groups further construct 0D clusters, 1D chains, 2D layers, or 3D frameworks, the rotation between the rigid B-O groups combined with expansion and/or tilting of other polyhedra in borate structures will control the thermal expansion property and may result in the anomalous thermal expansion. In recent years, many borate crystals have been reported to exhibit abnormal thermal expansion behaviors. For instance, the 1D NTE behavior has been detected in LiB_3_O_5_ [145] and BiB_3_O_6_ [146], the area NTE behaviors were discovered in LiBeBO_3_ [147] and KZnB_3_O_6_ [65,66], and the isotropic area NTE effect were found in KBBF [148]. Most interestingly, the 3D ZTE effect was discovered in Zn_4_B_6_O_13_ [67,68], which possess the intrinsic isotropic near-ZTE behavior as the first case. The discoveries of presented zincoborates add important members to the family of materials with anomalous thermal expansion properties. 

#### 3.3.1. Near-Zero Thermal Expansion Properties in Zn_4_B_6_O_13_

**Figure 14:**
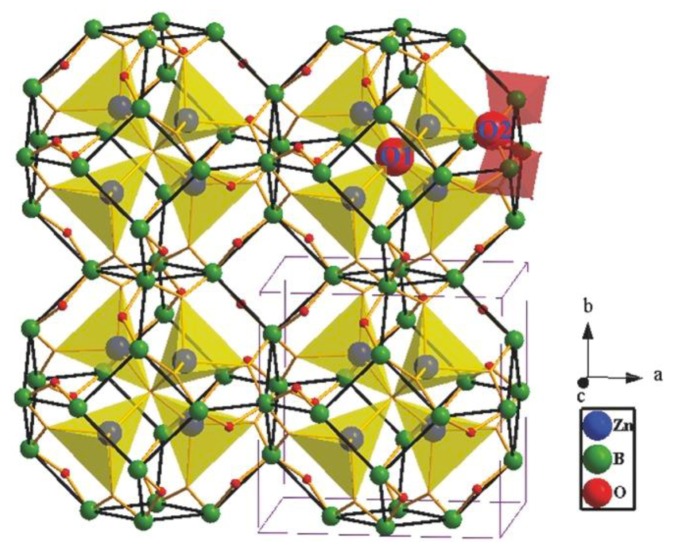
The crystal structure of Zn_4_B_6_O_13_. The neighboring boron atoms are connected by thick black lines to explicitly display the sodalite cages. Reprinted from Ref. [67], with permission of John Wiley and Sons.

Zn_4_B_6_O_13_ crystallizes in the cubic space group of *I*$\bar{4}$3*m* (No. 217) and possesses a very rare sodalite cage structure [67,68]. As shown in Figure 14, each [B_24_O_48_]^24−^ sodalite cage is constructed by 24 [BO_4_]^5−^ tetrahedra via sharing the corner oxygen atoms (O2 atoms). In detail, the [BO_4_]^5−^ tetrahedra are interconnected to form the quadrangles and hexagons with four and six [BO_4_]^5−^ units, respectively. Further, every six B4 quadrangles and eight B6 hexagons are combined to construct the closed [B_24_O_48_]^24−^ sodalite cage. The [Zn_4_O_13_]^18−^ cluster, locked in the [B_24_O_48_]^24−^ sodalite cage, is composed of four [ZnO_4_]^6−^ tetrahedra via sharing the vertex O1 atom located at the center of cage. The inside [Zn_4_O_13_]^18−^ cluster can effectively reinforce the [B_24_O_48_]^24−^ cage through the relatively strong Zn-O2 covalent bonds, which could decrease the thermal expansion.

**Figure 15:**
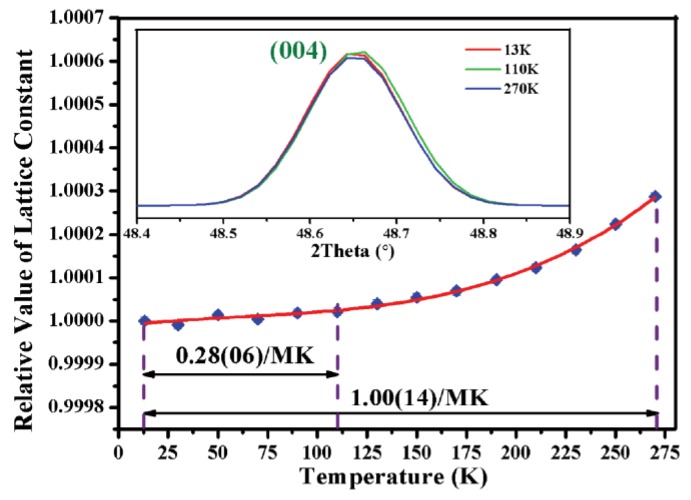
Variation of the refined cell parameters of Zn_4_B_6_O_13_ with respect to temperature. Reprinted from Ref. [67], with permission of John Wiley and Sons.

The thermal expansion behavior of Zn_4_B_6_O_13_ between 13 and to 270 K was investigated by the variable-temperature X-ray diffraction (XRD) and variation of refined cell parameters (refined by the Rietveld method). As results, in the measured temperature range, no new peaks appear in all the XRD patterns, which indicates that the structure of Zn_4_B_6_O_13_ is kept in the cubic *I*$\bar{4}$3*m* space group, and the thermal expansion is completely 3D isotropic. The cell parameter of Zn_4_B_6_O_13_ increases by just 0.03%, this thermal-expansion behavior is very low and consistent with the observation of positions of the XRD peaks, as shown in the insert in Figure 15, the (004) peaks remain nearly constant in the varying temperature environment. Further, the fitted average thermal expansion coefficient (by PASCal software) in the whole temperature range is 1.00(14)/MK. Particularly, from 13 to 110 K, the thermal expansion coefficient in Zn_4_B_6_O_13_ is even much smaller (0.28(06)/MK), which can be accurately cataloged to ZTE. 

First principles calculations were carried out for further investigation and demonstrate that the intrinsic isotropic near-ZTE behavior of Zn_4_B_6_O_13_ mainly originates from the invariability of the solid [B_24_O_48_]^24−^ cage fixed by the [Zn_4_O_13_]^18−^ clusters, affirming the important impact of the relatively strong Zn-O bonds. The discovery of Zn_4_B_6_O_13_ with intrinsic isotropic near-ZTE behavior not only gains an important member to the family of ZTE materials, but also revives the studies on new functionalities in borates, which may eventually lead to the discovery of more exciting and exotic emerging physicochemical properties in borates.

#### 3.3.2. Unidirectional Thermal Expansion in KZnB_3_O_6_

**Figure 16:**
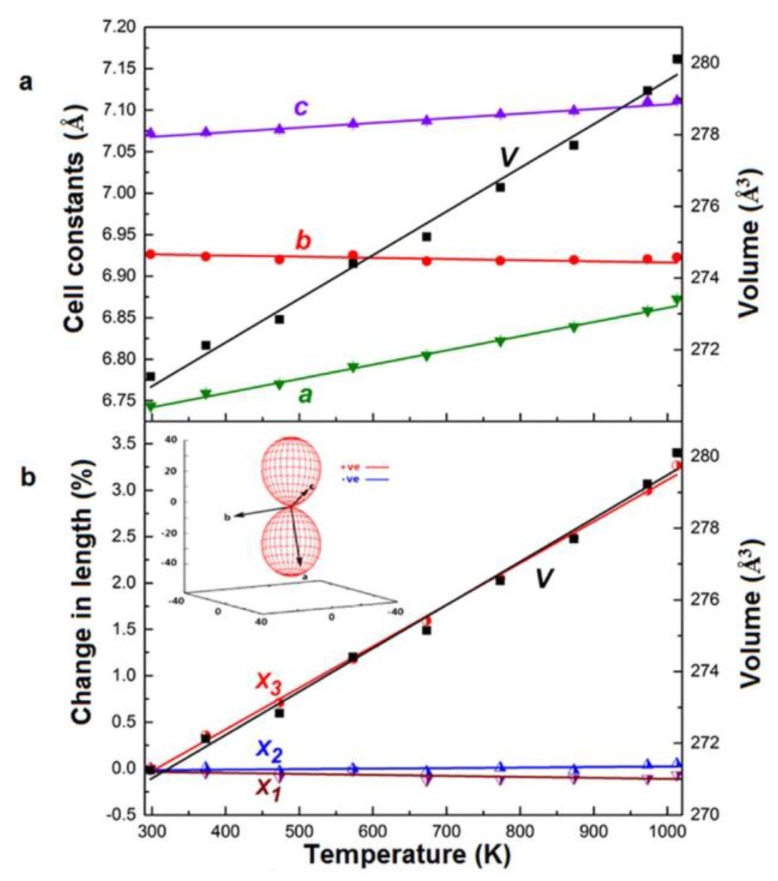
Thermal expansion behavior of the KZnB_3_O_6_. (**a**) The temperature dependence of lattice constants a, b, c and cell volume; (**b**) normalized components of the principal axes versus temperature. Reprinted with permission from Ref. [66], Copyright (2015) Creative Commons Attribution 4.0 International License.

As described before, KZnB_3_O_6_ is the first borate that contains the es-[BO_4_]^5−^ tetrahedra under ambient pressure [69,70]. Lou et al. investigated the thermal expansions of KZnB_3_O_6_ from room temperature to 1013 K (Figure 16a) [65,66]. Interestingly, KZnB_3_O_6_ shows an unusual unidirectional thermal expansion along the approximate [$\bar{3}$02] direction, i.e., the X_3_ axis direction, over the entire measured temperature (from 298 K to 1013 K). The expansions along other directions on the plane perpendicular to [$\bar{3}$02] are negligibly small, i.e., the area shows zero expansion (Figure 16b). Further investigations reveal that the abnormal thermal behavior originates from the cooperative hinge rotations of [B_6_O_12_]^6−^ (contain es-[BO_4_]^5−^ tetrahedra) and [Zn_2_O_6_]^8−^ rigid groups, which are probably driven by asymmetrical elongations of K-O bonds and only leads to a quasi-unidirectional expansion upon heating. These findings will help us better understand the relationship between structure and property and might broaden the applications of borates.

## 4. Single Crystal Growth of Zincoborates

High-quality and sizable single crystals are essential to measure fundamental properties and to accurately evaluate practical applications. Although a great deal of effort has been put into the exploration of growing sizable single crystals with high optical quality, it is still a great challenge to obtain the large-scale crystals for practical devices [149,150,151]. As for the zinc-containing system, the growth of large crystal seems more difficult since compounds containing zinc element usually have a high melting point (ZnO, 1975 °C at 5.2 MPa). Usually, the effective fluxes, such as PbO, PbF_2_, H_3_BO_3_, etc., are introduced to decrease the melting point and the viscosity during the growth of single crystals. Fortunately, some of them melt congruently and sizable crystals have been grown from a stoichiometric melt by the top-seeded solution growth (TSSG) and Czochralski method.

### 4.1. Bi_2_ZnOB_2_O_6_

**Figure 17:**
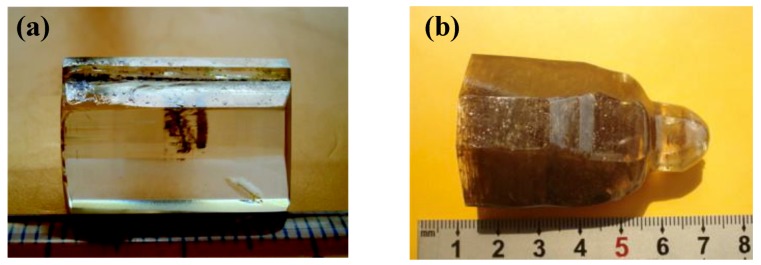
(**a**) Photograph of Bi_2_ZnOB_2_O_6_ crystal grown by the top-seeded solution growth (TSSG) method and (**b**) Czochralski method, respectively. Reprinted with permission from Ref. [83,152], Copyright (2009) American Chemical Society, Copyright (2010) Elsevier, respectively.

In 2009, Li et al. successfully grew the single crystal of Bi_2_ZnOB_2_O_6_ with high quality and dimensions of 18 mm × 13 mm × 6 mm through the TSSG method (Figure 17a) [83]. The low melt point (no more than 700 °C), non-viscous properties, and the congruent melting performance make Bi_2_ZnOB_2_O_6_ capable to grow sizable single crystals. Followed by these, a sizable single crystal with sizes up to Φ 30 mm × 55 mm has been obtained along the *c*-axis direction using the Czochralski method (Figure 17b) [152]. The NLO coefficients have been determined through the Maker fringes method at 1064 nm [140]. Results show that the coefficients of Bi_2_ZnOB_2_O_6_ relative to *d*_36_ for KDP are *d*_31_ = (2.34 ± 0.05) *d*_36_ (KDP), *d*_32_ = (7.90 ± 0.16) *d*_36_ (KDP) and *d*_33_ = (2.60 ± 0.06) *d*_36_ (KDP). The large NLO coefficients and the easy crystal growth behavior suggest that Bi_2_ZnOB_2_O_6_ is a promising candidate for NLO materials.

### 4.2. Ba_3_(ZnB_5_O_10_)PO_4_

**Figure 18:**
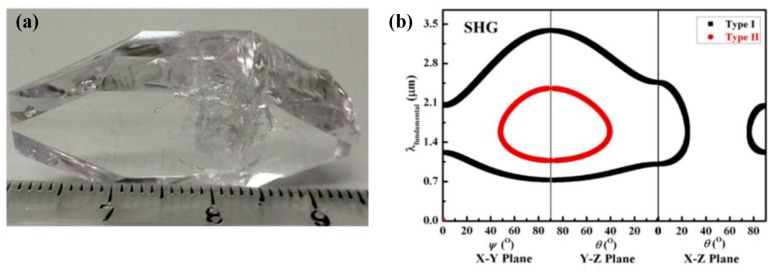
(**a**) The as-grown Ba_3_(ZnB_5_O_10_)PO_4_ crystal with [101]-oriented seed; (**b**) Phase-matching calculation for Ba_3_(ZnB_5_O_10_)PO_4_ crystal: PM angle curves for type-I (black) and type-II (red) SHG as a function of the fundamental wavelength. Reprinted (adapted) with permission from Ref. [153], Copyright (2016) American Chemical Society.

Ba_3_(ZnB_5_O_10_)PO_4_ melts congruently, but its relatively high viscosity and melting temperature are unfavorable to obtain high-quality crystals from a stoichiometric melt. Therefore, large single crystals of Ba_3_(ZnB_5_O_10_)PO_4_ were grown through a TSSG method by using the ZnO-B_2_O_3_ self-flux system (Figure 18) [153]. The seed orientations have a great impact on the growth rate, morphology, and quality of the crystals. With the [010]- and [101]-oriented seed, Ba_3_(ZnB_5_O_10_)PO_4_ crystals with size dimensions of 35 mm × 20 mm × 5 mm and 34 mm × 15 mm × 8 mm were obtained, respectively, both of which have high optical quality. Compared with the crystal grown with the [010]-oriented seed, the crystal grown with the [101]-oriented seed has a thicker dimension and exhibits a more regular shape (Figure 18a). Refractive index measurements show that Ba_3_(ZnB_5_O_10_)PO_4_ is a negative biaxial crystal with birefringence ranging from 0.0418 to 0.0306 in a wavelength range of 253.6–2325.4 nm. Based on the measured refractive index and fitted Sellmeier equations, the calculated phase matching (PM) regions for SHG based on the fundamental light are 730–3386 nm for type I SHG PM (Figure 18b). The measurement results indicate that Ba_3_(ZnB_5_O_10_)PO_4_ crystal is a promising NLO material in the UV region.

### 4.3. β-Zn_3_BPO_7_

**Figure 19:**
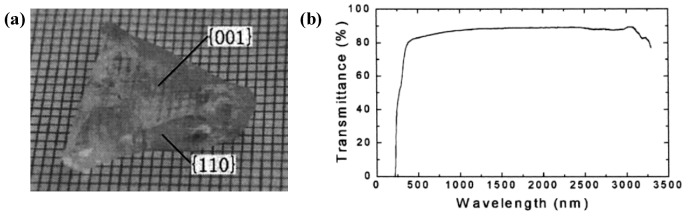
(**a**) Bottom of the as-grown *β*-Zn_3_BPO_7_ crystal with size up to 43 mm × 43 mm × 12 mm; (**b**) transmittance spectrum of *β*-Zn_3_BPO_7_. Reprinted with permission from Ref. [136], Copyright (2002) American Chemical Society.

In 1982, Liebertz and Stahr reported the existence of Zn_3_BPO_7_ that occur in two phases with a phase transition at 602 °C [154]. *β*-Zn_3_BPO_7_ (high-temperature phase) has been characterized as a NLO crystal owning to its significant properties. However, the growth of large crystals of *β*-Zn_3_BPO_7_ is difficult since the crystal will transfer from *β*- to *α*-phase. Unremitting efforts have been made to obtain sizable and high-quality *β*-Zn_3_BPO_7_ crystals. Consequently, the phase transition of *β*-Zn_3_BPO_7_ to *α*-Zn_3_BPO_7_ is effectively suppressed through adopting appropriate heat treatment. In 2000 to 2002 [136,155,156], the transparent and crack free single crystals of *β*-Zn_3_BPO_7_ with size dimensions of 35 mm × 20 mm × 10 mm and 43 mm × 43 mm × 12 mm (Figure 19a) were grown by Wu and Wang et al. using the Czochralski and TSSG methods, respectively. 

The linear and non-linear optical properties are investigated. Results show that *β*-Zn_3_BPO_7_ has a UV absorption edge at about 250 nm (Figure 19b) and the none-zero NLO coefficient *d*_11_ measured by the Maker fringes method is 0.69 pm/V (1.8 times as large as that of *d*_36_ (KDP)). The Sellmeier equations suggest that the shortest SHG wavelengths for the crystal are 399 and 605 nm for types I and II, respectively. The easy growth habit and good NLO properties make *β*-Zn_3_BPO_7_ attractive for continued research as NLO materials. 

### 4.4. Zn_4_B_6_O_13_

**Figure 20:**
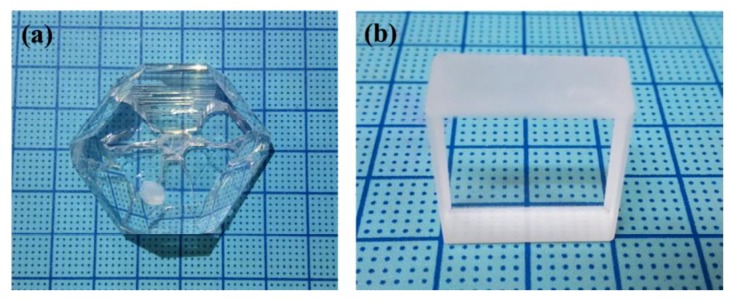
(**a**) The as-grown Zn_4_B_6_O_13_ single crystal with a dimension of 40 mm × 40 mm × 18 mm; (**b**) Fabricated single crystal with a dimension of 20 mm × 20 mm × 10mm. Reprinted from Ref. [67], with permission of John Wiley and Sons.

The large-sized Zn_4_B_6_O_13_ single crystal with dimensions of about 40 mm × 40 mm × 18 mm and exhibiting good optical quality was grown using the conventional TSSG method (Figure 20) [67]. Optical transmittance measurements show that Zn_4_B_6_O_13_ possesses a wide transmission range covering a wide spectral region from the UV to the near-infrared (wavelength from 217 to 3100 nm). The UV cutoff edge of Zn_4_B_6_O_13_ is the shortest among the ZTE crystals, implying the potential applications of Zn_4_B_6_O_13_ in ultra precise optical instruments. Notably, the short UV cutoff edge of Zn_4_B_6_O_13_ also stems from the relatively strong Zn-O bond based on the analysis of the ab initio partial density of states. Moreover, Zn_4_B_6_O_13_ exhibits high thermal stability, thermal conductivity and high mechanical hardness, which are also important for practical applications. Combined with the intrinsic isotropic near-ZTE behavior, the environmentally friendly feature and easy growth habit facilitate the practical applications of Zn_4_B_6_O_13_.

### 4.5. BaZnBO_3_F

**Figure 21:**
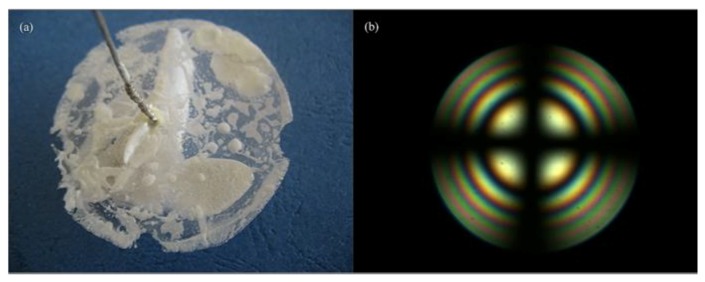
(**a**) The as-grown BaZnBO_3_F crystal; (**b**) Interference pattern of BaZnBO_3_F along the *c*-axis. Reprinted from Ref. [135], Copyright (2016) with permission from Elsevier.

BaZnBO_3_F exhibits typical layer habit due to structural characteristics, which is familiar with the KBBF family crystals. Crystal of BaZnBO_3_F with the dimensions of about 20 mm × 20 mm × 0.5 mm has been grown by high temperature solution method from BaF_2_-NaF flux (Figure 21) [135]. The perfect coplanar and alignment [BO_3_]^3−^ groups in the structure result in an observed large effective NLO coefficient (2.8 × *d*_eff_ KDP). BaZnBO_3_F crystal possesses chemical stability and high transmittance in the range of 300–3000 nm wavelength with the UV cut-off edge of 223 nm. In the consideration of its superior optical properties in the visible to UV range, larger-size crystals should be developed for practical applications by exploiting several methods or flux to overcome the strong anisotropic growth habit. 

## 5. Conclusions

In this review, we have examined the recent development of zincoborates, focusing on the crystal structure chemistry as well as their physicochemical properties. The introduction of the strong-bonded zinc cations into borates effectively enriches the structural diversity of borates and further results in extensive applications. Several examples were given. (1) A series of zincoborates display unique structural features in crystal chemistry of borates, for example, KZnB_3_O_6_ and Ba_4_Na_2_Zn_4_(B_3_O_6_)_2_(B_12_O_24_) with novel es-[BO_4_]^5^^−^ tetrahedra; Ba_4_K_2_Zn_5_(B_3_O_6_)_3_(B_9_O_19_), Ba_2_KZn_3_(B_3_O_6_)(O(B_3_O_6_)_2_) with two kinds of isolated polyborate anionic groups coexisting in one borate structure; AZn_2_BO_3_X_2_ (A = Na, K, Rb, NH_4_; X = Cl, Br) series, Cs_3_Zn_6_B_9_O_21_, BaLiZn_3_(BO_3_)_3_ with benign KBBF-type layered structures, etc. (2) Numerous zincoborates with brilliant physicochemical performance have emerged. For instance, Cs_3_Zn_6_B_9_O_21_, BaZnBO_3_F, Bi_2_ZnOB_2_O_6_, Ba_5_Zn_4_(BO_3_)_6_, Ba_3_(ZnB_5_O_10_)PO_4_, etc. have been suggested to be suitable for UV NLO applications; Zn_4_B_6_O_13_ and KZnB_3_O_6_ with anomalous thermal expansion properties have inspired the discovery of different applications for borates. 

Based on the aforementioned findings, the regulation effect of introducing Zn-O/F polyhedra into borates can be emphasized. Firstly, the introduction of Zn-O/F polyhedra can effectively inhibit the polymerization of B-O anionic structures, which is beneficial to obtain isolated B-O groups. In particular, it is propitious to obtain good NLO or birefringent properties when the isolated B-O groups are induced by Zn-O/F polyhedra and exhibit a coplanar arrangement. The terminal oxygen atoms of the B-O groups are linked with zinc atoms, eliminating the dangling bonds of the B-O groups, which would further widen the transparence in the UV region. Moreover, the distorted [ZnO_4_]^6−^ and [ZnO_3_F]^5−^ tetrahedra are NLO-active structural units, which should provide an enhanced contribution to the SHG response, indicating that the zinc-containing borate system is optimal for exploring new NLO materials. However, it is not yet clearly understood which factors determine the special effect of Zn-O/F polyhedra in zincoborates. The intrinsic mechanism understanding of the special contribution of the covalent zinc cations on structural and functional regulation should be theoretically elucidated and exploited in the future, which will present an useful guide for the exploration of undiscovered NLO crystals in zincoborate system that can be practically applied for UV/DUV NLO materials. 

In addition, although several zincoborates have been grown with sizable single crystals, there are still great hurdles to develop new zincoborates with excellent properties that are feasible for growing large single crystals. Looking into the future, continuous exploration and considerable effort should be made in growing large-size single crystals for more detailed physical measurements and practical applications.

## Figures and Tables

**Table 1:** The representative non-linear optical (NLO) zincoborates.

Compounds	Space Group	Structural Features	Second Harmonic Generation (SHG) Intensity (@ 1064nm)	Absorption Edge	Refs.
Cs_3_Zn_6_B_9_O_21_	*Cmc*2_1_	^2^_∞_[Zn_2_BO_3_O_2_] layer	3.3 × KH_2_PO_4_ (KDP)	~200 nm	[45]
KZn_2_BO_3_Cl_2_	*R*32	Isolated [BO_3_]^3−^ (coplanar)	3.01 × KDP	~194 nm	[58]
RbZn_2_BO_3_Cl_2_	*R*32	Isolated [BO_3_]^3−^ (coplanar)	2.85 × KDP	~198 nm	[58]
NH_4_Zn_2_BO_3_Cl_2_	*R*32	Isolated [BO_3_]^3−^ (coplanar)	2.82 × KDP	~190 nm	[58]
KZn_2_BO_3_Br_2_	*R*32	Isolated [BO_3_]^3−^ (coplanar)	2.68 × KDP	~209 nm	[58]
RbZn_2_BO_3_Br_2_	*R*32	Isolated [BO_3_]^3−^ (coplanar)	2.53 × KDP	<214 nm	[58]
Ba_3_(ZnB_5_O_10_)PO_4_	*Pmn*2_1_	/	4 × KDP (@ 532nm)	~180 nm	[60]
Ba_5_Zn_4_(BO_3_)_6_	*Pc*	^2^_∞_[Zn_4_(BO_3_)_4_O_6_] layer	2.6 × KDP	~223 nm	[62]
Ba_2_Zn(BO_3_)_2_	*Pca*2_1_	Isolated [BO_3_]^3−^	1.5 × KDP	~230 nm	[64]
Bi_2_ZnOB_2_O_6_	*Pba*2	Isolated [B_2_O_5_]^4−^ + [B_2_O_7_]^8−^	3–4 × KDP	~330 nm	[83]
*α*−Pb_2_Ba_4_Zn_4_B_14_O_31_	*P*1	Isolated [B_2_O_5_]^4−^ + [B_6_O_13_]^8−^	0.6 × KDP	<289 nm	[84]
*β*−Pb_2_Ba_4_Zn_4_B_14_O_31_	*Cc*	Isolated [B_2_O_5_]^4−^ + [B_6_O_13_]^8−^	1.1 × KDP	<304 nm	[84]
Cs_12_Zn_4_(B_5_O_10_)_4_		^2^_∞_[Zn(B_5_O_10_)] layer	0.5 × KDP	<185 nm	[130]
BaZnBO_3_F	*P* $\bar{6}$	Isolated [BO_3_]^3−^ (coplanar)	2.8 × KDP	~223 nm	[135]
*β*-Zn_3_BPO_7_	*P* $\bar{6}$	/	1.8 × KDP	~250 nm	[136]
Mg_2_Na_2_ZnB_4_O_10_	*/*	/	2.78 × KDP	~210 nm	[137]
